# Executive functions, impulsivity, and inhibitory control in adolescents: A structural equation model

**DOI:** 10.5709/acp-0154-5

**Published:** 2014-05-15

**Authors:** Emanuele Fino, Sergio Melogno, Paolo Iliceto, Sara D’Aliesio, Maria Antonietta Pinto, Gabriella Candilera, Ugo Sabatello

**Affiliations:** 1 Department of Developmental and Social Psychology, Sapienza University of Rome, Italy; 2 Department of Pediatrics and Child Neuropsychiatry, Sapienza University of Rome, Italy; 3 CEO of S&P Statistics and Psychometrics Ltd, Rome, Italy; 4 Clinical Psychologist, Private Practice, Rome, Italy

**Keywords:** executive function, impulsivity, inhibitory control, sensation seeking, personality

## Abstract

**Background**. Adolescence represents a critical period for brain development, addressed by neurodevelopmental models to frontal, subcortical-limbic, and striatal activation, a pattern associated with rise of impulsivity and deficits in inhibitory control. The present study aimed at studying the association between self-report measures of impulsivity and inhibitory control with executive function in adolescents, employing structural equation modeling. **Method**. Tests were administered to 434 high school students. Acting without thinking was measured through the Barratt Impulsiveness Scale and the Dickman Impulsivity Inventory, reward sensitivity through the Behavioral Activation System, and sensation seeking through the Zuckerman–Kuhlman–Aluja Personali- ty Questionnaire. Inhibitory control was assessed through the Behavioral Inhibition System. The performance at the Wisconsin Card Sorting Task indicated executive function. Three models were specified using Sample Covariance Matrix, and the estimated parameters using Maximum Likelihood. **Results**. In the final model, impulsivity and inhibitory control predicted executive function, but sensation seeking did not. The fit of the model to data was excellent. **Conclusions**. The hypothesis that inhibitory control and impulsivity are predictors of executive function was supported. Our results appear informative of the validity of self-report measures to examine the relation between impulsivity traits rather than others to regulatory function of cognition and behavior.

## Introduction

Adolescence represents a critical period for brain development. Age-related changes include alterations in sensitivity to salient stimuli, addressed by neurodevelopmental models to enhanced frontal, subcortical-limbic, and striatal activation, a pattern associated with the rise of impulsivity (IMP) and deficits in inhibitory control (IC; Romer et al., 2009), marking the risk for psychopathology and maladaptive behaviors.

Although several conceptualizations of IMP have been proposed, it is generally defined as the tendency to fast, spontaneous, unplanned, and potentially maladaptive reaction to environmental cues. Findings from prior research indicate that IC (i.e., the ability to suppress responses) underlies IMP; neuroimaging studies show that IMP and IC are regulated by the function of the prefrontal cortex (PFC), one of the last regions of human brain to reach structural and functional maturation (Horn, Dolan, Elliott, Deakin, & Woodruff, 2003).

In fact, protracted pruning of the PFC denotes growing control over behavior (Romer, 2010), and delays in the development of the PFC explain adolescents’ observed IMP and poor IC. In particular, three cortical areas appear to be implicated in this pattern: the ventromedial prefrontal cortex, the orbitofrontal cortex, and the dorsolateral prefrontal cortex (Bechara, 2005).

These areas are responsible for a set of supervisory cognitive processes, regulating cognitive activity, emotional response, and overt behavior, defined as *executive function* (EF; Anderson, 1998). Interference control, cognitive and behavioral inhibition represent a set of abilities related to EF, defined by Nigg (2000, p. 237) as “executive inhibition”. These are evident in situations that require a fast cognitive and behavioral adjustment to novel or shifting requests of the environment.

Neuropsychological research shows that EF has an extended course of development (Diamond, 2002). Consistently, variations in IC and IMP are observed from early childhood, continuing into adolescence (Williams, Ponesse, Schachar, Logan, & Tannock, 1999). In particular, adequate EF is associated with the performance at the Wisconsin Card Sorting Task (WCST; Grant & Berg, 1948; Heaton, 1993), a valid test in assessing the ability to shift cognitive set in response to changing rules. A commonly used indicator of WCST performance is *perseveration*, which is defined as the persistence in responding to a previous, but currently no longer relevant, sorting principle.

Individual variations in impulse control observed in adolescents are linked to IMP traits that develop early, and several classifications have been reported in the literature. However, evidence from self-report (Miller, Joseph, & Tudway, 2004) and neuroimaging studies (Romer et al., 2009) supports a three-independent-component structure of IMP, including the traits of *acting without thinking, reward sensitivity*, and *novelty seeking*. The assessment of IMP through self-reports is considered as particularly difficult given the ambiguity surrounding the construct and the availability of different measures. Nevertheless, as highlighted in previous studies, this is an important research question because of the need for accurate risk assessment in clinical and forensic populations (Miller et al., 2004, p. 351), particularly aiming to address specific facets of the construct.

Acting without thinking represents the tendency to behave without premeditation and forethought in response to environmental stimuli in demanding or stressing situations. Research showed that this form of IMP is associated with EF, in particular, with the ability to manage conflicting environmental requests and inhibit responses when these are no longer functional. This trait is measured by at least two self-report scales: The Barratt Impulsivity Scale (BIS-11; Patton, Stanford, & Barratt, 1995), especially through the motor and non-planning subscales (Romer, 2010), and the dysfunctional impulsivity scale included in the Dickman Impulsivity Inventory (DII; Dickman, 1990).

Reward sensitivity is characterized by the urge to accept an immediate and less significant reward rather than delaying in favor of a more meaningful reward, reflecting enhanced predisposition to boredom. A valid measure of reward sensitivity is the BIS/BAS developed by Carver and White (1994) on the basis of Gray’s (1981) neuropsychological theory of personality. According to this last model, two mechanisms account for individual variations in two major personality dimensions: The Behavioral Activation System (BAS), related to cognitive activation, and the Behavioral Inhibition System (BIS), related to IC. Although divided into three subscales (Fun, Drive, Reward), BAS was found to significantly load onto a single dimension (Miller et al., 2004), suggesting a single BAS component related to impulse control, opposing to the BIS.

Finally, the discussion on IMP in terms of a general model of personality (Whiteside & Lynam, 2001) allowed identifying the *novelty seeking* trait, defined as the desire for varied, novel, complex, and intense sensations and experiences. The sensation seeking (SS) dimension, included in the Five-Factor Model of Personality (Aluja, Kuhlman, & Zuckerman, 2010), is assessed through the Zuckerman-Kuhlman-Aluja Personality Questionnaire (ZKA-PQ). Nevertheless, Aslan and Cheung-Blunden (2012) recently showed that EF-related factors were not significantly linked to dimensions assessed through the Five-Factor Model of Personality, suggesting that more work is needed to better understand such relations.

However, Romer (2010) showed that the three above IMP traits, although positively related to age, are not equally associated with EF. Individual differences in the activation of the dorsal and ventral striatum, responsible for impulse regulation and explaining adolescents’ early forms of risky behaviors, determine a negative relation between acting without thinking and EF and between reward sensitiveness and EF, but novelty seeking was found to be even positively related to working memory abilities (Romer et al., 2009). In Romer’s neurodevelopmental model, risk-taking observed during adolescence can be considered as the outcome of developmental concerns, attributed more to a lack of experience than to structural impairment in frontal control.

Nevertheless, although prior research has provided several psychometric examinations of most widely used self-report measuresof IMP and their underlying factor structure (Miller et al., 2004; Whiteside & Lynam, 2001), to date, few studies have examined the pattern of relations between IMP traits measured by self-report scales and EF. This is so, albeit self-report measures revealed valid in assessing IMP and allowed to avoid the mediation of factors potentially interfering with the assessment of IMP, for instance, autonomic arousal, as observed in laboratory tasks (see Enticott, Ogloff, & Bradshaw, 2006).

In this study, by means of a structural model, we aimed at studying the relation of self-report measures of IMP, IC, and personality to EF in adolescents. We hypothesized that high scores at the BIS-11 and the DII (measures of acting without thinking); high scores at the BAS; and conversely, low scores at the BIS (measures of reward sensitivity and of IC), respectively, predict adolescents’ EF and related frontal maturation, resulting in high perseverative performances at the WCST, while SS (measure of novelty seeking) do not, in line with findings from neurodevelopmental research (Romer, 2010; Romer et al., 2009).

## Method

### Participants

After obtaining the permission from managers of the schools, between August and October 2012, we administered the psychometric tests to 434 high school students from the North of Italy. The participants were aged 16 to 18 years, 229 males (*M*_age_ = 17.07, *SD* = 0.82) and 205 females (*M*_age_ = 16.94, *SD* = 0.86). No differences were found between the age of the females and the age of the males, *t*(432) = 1.53, *p* = .12. The subjects participated voluntarily in the study, and each subject provided written informed consent. The study protocol received ethics approval from the local research ethics review board.

### Instruments

The Barratt Impulsiveness Scale (11th version, BIS-11; Patton et al., 1995) consists of a short questionnaire designed to measure IMP. It contains 30 items, each of which is answered on a 4-point Likert scale (*rarely/never* = 1, *occasionally* = 2, *often* = 3, *almost always/always* = 4), and the level of IMP is calculated by summing up the scores for each item. The second-order factor analysis for the six primary factors identified three components as follows: (a) Cognitive, (b) Motor, and (c) Non-Planning. The Italian version of the questionnaire has demonstrated good reliability (Cronbach’s α = .79 for Total IMP) and validity (Fossati, Di Ceglie, Acquarini, & Barratt, 2001).

The Behavioural Inhibition System and Behavioural Activation System (BIS/BAS; Carver & White, 1994) is a 20-item test using two 4-point scales (1 = *strongly disagree* to 4 = *strongly agree*) designed to assess dispositional sensitivity to the Behavioural Inhibition System (BIS) and the Behavioural Activation System (BAS), respectively. Moreover, BAS is composed of three sub-scales of its own: (a) Reward Responsiveness, (b) Drive, and (c) Fun Seeking. In the present sample, internal consistencies were satisfactory (Cronbach’s α = .77 for BIS, and .82 for total BAS).

The Dickman Impulsivity Inventory (DII; Dickman, 1990) is a self-report questionnaire developed to measure two types of IMP, namely, Functional and Dysfunctional IMP. It consists of 23 items with a true/false answer format. Eleven items are designed to tap functional IMP, while another 12 items tap dysfunctional IMP. *Dysfunctional* IMP is defined as the tendency to act with less forethought than most people of equal ability. *Functional* IMP, in contrast, is the tendency to act with relatively little forethought when such a style is optimal. Cronbach’s alpha was .81 for Dysfunctional IMP, and .78 for Functional IMP.

The Wisconsin Card Sorting Task (WCST; Heaton, 1993) is considered to be a prototype of a task assessing abstract reasoning by frontal lobe function in adolescent or adult populations, because it addresses the ability to conceptualize abstract categories, apply detected concepts, and shift the cognitive set according to changing contingencies. WCST is one the most used experimental tasks to assess EF. In WCST, participants are asked to infer, by trial and error, with minimum feedback, a relevant sorting rule out of three possible sorting rules (i.e., the color, shape, or number of the stimuli). After 10 correct sorts, the sorting rule changes without warning, requiring participants to find the newly relevant sorting rule (Heaton, 1993). A commonly used indicator of WCST performance is *perseveration*, which is defined as the persistence in responding to a previous, but currently no longer relevant, sorting principle. After a pattern of correct sorting is established, the rule changes and the participant must adjust to the change. In fact, the error pattern in WCST performance seems to reflect the relation between neuropsychological dysfunction and IMP. In the Italian validation sample, Cronbach’s alpha ranged from .69 to .84 (Laiacona, Inzaghi, De Tanti, & Capitani, 2000).

The Zuckerman-Kuhlman-Aluja Personality Questionnaire (ZKA-PQ; Aluja et al., 2010) is a 200-item questionnaire based on the theoretical constructs of the alternative Five-Factor Model of Personality. The instrument measures Aggressiveness (physical and verbal aggression, anger, hostility), Activity (work compulsion, general activity, restlessness, work energy), Extraversion (positive emotions, social warmth, exhibitionism, sociability), Neuroticism (anxiety, depression, dependency, low self-esteem), and SS (thrill and adventure seeking, experience seeking, disinhibition, boredom; susceptibility/IMP). The authors reported that Cronbach’s alphas were .78 to .81, .76 to .73, .75 to .75, .74 to .79, and .70 to .72, respectively.

### Statistical analysis

Two-tailed *t*-tests were used for continuous variables using SPSS 17.0 (SPSS Inc., Chicago, IL, USA). We examined the hypothesized relations in the model by using LISREL 8.30.

SEM relies on several statistical tests to determine the adequacy of model fit to the empirical data, taking into account the modeling of multiple latent independents, each measured by multiple indicators, and one or more latent dependents, as well measured with multiple indicators. The process centers on two steps: validating the measurement model and fitting the structural model. This starts by specifying a model on the basis of predefined theory and results from prior empirical research, and two or more alternative models are then compared in terms of model fit. Consistently, it is possible to measure the extent to which the covariances predicted by the model correspond to the observed covariances in the data, by means of the statistical fitting of the factor model to the observed data (variances and co-variances or correlations), the assessment of fit, and the interpretation of the results.

We used the following criteria to evaluate the overall goodness-of-fit. The χ^2^ value close to zero indicates little difference between the expected and observed covariance matrices, with the probability level greater than .05 evidencing the absence of meaningful unexplained variance. Moreover, to estimate a better goodness of fit, due to the fact that χ^2^ is sensitive to sample size, we calculated the ratio of χ^2^ to degrees of freedom that should be less than 3 as an acceptable data-model fit. In addition to the χ^2^/*df* test, we utilized the Goodness-of-fit Index (GFI), the Comparative Fit Index (CFI), the Root Mean Square Error of Approximation (RMSEA), and the Standardized Root Mean Square Residual (SRMR). Indicators of a well-fitting model are evidenced by GFI and CFI greater than .95, RMSEA less than .06 and SRMR less than .08.

## Results

We conducted a preliminary study taking gender into account, because given the age of the subjects, the frontal cortex development in females is often ahead of that in males, and gender might therefore produce significant differences in EF. Accordingly, we compared males and females on all the scales, but no statistically significant difference was found (Table 1).

**Table 1.** Comparisons Between Subjects

Scales	Males (*n* = 229)	Females (*n* = 205)	*t*(432)	*p*
Errors (WCST)	7,97 ± 3,02^a^	8,38 ± 3,22^a^	1,34	.16
Perseverative Errors (WCST)	6,90 ± 2,11^a^	7,27 ± 2,47^a^	1,67	.09
Behavioural Inhibition System (BIS)	18,90 ± 4,65^a^	19,11 ± 4,91^a^	0,46	.64
Behavioural Activation System (BAS)	29,25 ± 4,14^a^	28,72 ± 4,55^a^	1,27	.20
Impulsivity (BIS-11)	59,38 ± 8,32^a^	57,60 ± 5,83^a^	1,48	.13
Dysfunctional Impulsivity (DII)	2,03 ± 1,29^a^	1,89 ± 1,27^a^	1,11	.26
Aggression (ZKA-PQ)	99,06 ± 20,45^a^	98,32 ± 20,63^a^	0,37	.70
Activity (ZKA-PQ)	105,88 ± 11,58^a^	104,95 ± 11,42^a^	0,84	.40
Extraversion (ZKA-PQ)	117,83 ± 15,17^a^	118,60 ± 15,31^a^	0,53	.59
Neuroticism (ZKA-PQ)	91,24 ± 19,43^a^	90,63 ± 19,64^a^	0,32	.74
Sensation Seeking (ZKA-PQ)	97,49 ± 18,94^a^	96,40 ± 18,51^a^	0,60	.54

*Note*. BIS-11 = the Barratt Impulsivity Scale (Patton, Stanford, & Barratt, 1995). DII = the Dickman Impulsivity Inventory (Dickman, 1990). WCST = the Wisconsin Card Sorting Task (Grant & Berg, 1948). ZKA-PQ = the Zuckerman–Kuhlman–Aluja Personality Questionnaire (Aluja, Kuhlman, & Zuckerman, 2010). ^a^ Values shown as mean ± standard deviation.

In order to proceed with SEM and maximum likelihood estimation, we tested for the normality of the scales. Given that, in normal distributions, skewness and kurtosis should be comprised within 2 and +2 range, we assumed the data as normally distributed. The descriptive statistics of all the scales (minimum, maximum, mean, standard deviation, skewness, and kurtosis) are listed in Table 2.

**Table 2.** Descriptive Statistics of the Scales

Scales	Min	Max	*M*	*SD*	Kurtosis	Skewness
Errors (WCST)	4	13	8,16	3,12	-1,394	0,535
Perseverative Errors (WCST)	3	12	7,07	2,29	0,400	1,182
Behavioural Inhibition System (BIS)	10	34	19,00	4,77	0,101	0,691
Behavioural Activation System (BAS)	20	49	29,00	4,34	1,661	0,754
Impulsivity (BIS-11)	3	94	58,53	12,44	1,24	-1,529
Dysfunctional Impulsivity (DII)	0	4	1,96	1,28	-1,106	0,027
Aggression (ZKA-PQ)	49	114	98,71	20,54	.875	-1,526
Activity (ZKA-PQ)	81	120	105,44	11,5	-.288	-0,830
Extraversion (ZKA-PQ)	83	142	118,20	15,14	.252	-0,662
Neuroticism (ZKA-PQ)	56	121	90,95	19,51	-.994	-0,384
Sensation Seeking (ZKA-PQ)	62	125	96,97	18,72	-.665	-0,523

*Note*. BIS-11 = the Barratt Impulsivity Scale (Patton, Stanford, & Barratt, 1995). DII = the Dickman Impulsivity Inventory (Dickman, 1990). WCST = the Wisconsin Card Sorting Task (Grant & Berg, 1948). ZKA-PQ = the Zuckerman–Kuhlman–Aluja Personality Questionnaire (Aluja, Kuhlman, & Zuckerman, 2010).

Three models were specified, using the Sample Covariance Matrix and the estimated parameters using Maximum Likelihood. In the first model, we aimed at testing the relations between Personality (Aggressiveness, Activity, Extraversion, and Neuroticism), IMP (BIS-11, DII, SS), and Control (low BIS and high BAS) as predictors of EF (poor performance at the WCST). All of the factors were allowed to correlate. The model produced fit indices as follows: χ^2^(38) = 180.66 (*p* < .000); χ^2^/*df* = 4.75; CFI = .82; RMSEA = .09; SRMR = .06. As expected, we found moderate correlations between factors and irrelevant contributions of Personality to EF. Particularly, Aggressiveness and Neuroticism showed inadequate statistical significance. Thus, this model was rejected. In the second model, Aggressiveness and Neuroticism were excluded from Personality, but - as expected - also this model was weak, producing fit indices as follows: χ^2^(21) = 126.72 (*p* < .000); χ^2^/*df* = 6.03; CFI = .85; RMSEA = .11; SRMR = .06. In the final model, we tested IMP and Control as predictors of EF. Personality and SS were not included. The fit of the model to the data was excellent: χ^2^(6) = 9.82 (*p* = .13); χ^2^/*df* = 1.64; CFI = .99; RMSEA = .03; SRMR = .02, showing that the Five-Factor Model of Personality was not significantly linked to EF, in line with our theoretical assumptions.

**Figure 1.**
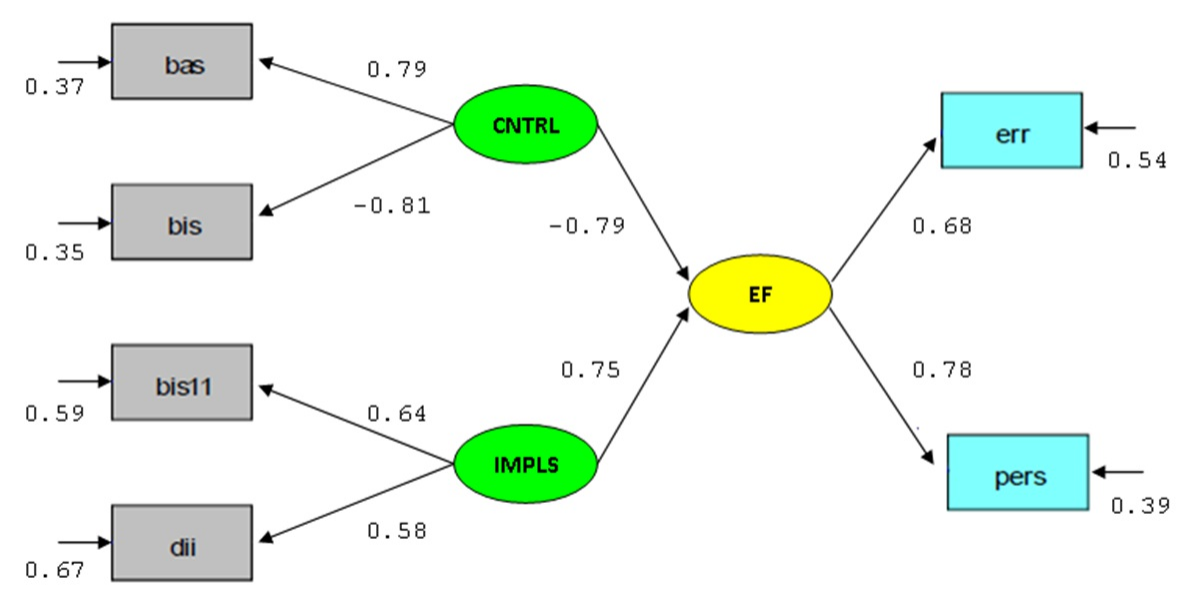
Structural model. Bas = Behavioural Activation System (BAS). Bis = Behavioural Inhibition System (BIS). Bis11 = Impulsivity (BIS-11). CNTRL = Control. Dii = Dysfunctional Impulsivity (DII). EF = Executive Function. Err = Errors (WCST). IMPLS = Impulsivity. Pers = Perseverative.

In the measurement model, the factor loadings that accompany each arrow in the model (Figure 1) represent the strength of the relationship between the variables, and they all were high, above .50, and statistically significant. In terms of squared multiple correlation coefficients (*R*^2^) that describe the amount of variance the common factor accounts for in the indicator variables, the latent variable Control explains about 65% of the variance of BIS and 63% of BAS, IMP explains about 41% of the variance of BIS11 and 33% of DII, while EF explains about 46% of the errors and 61% of the perseverative errors of the WCST. Eventually, both the latent predictor factors, Control and IMP, significantly explain EF (structural regression coefficients = -.79 and .75, respectively), with about 71% of variance.

## Discussion

In the present study, we examined the relation of IMP and IC to EF. Results from SEM confirmed the adequacy of a theoretical model in which acting without thinking and poor behavioral inhibition predicted EF in adolescents, highlighting that only specific IMP and IC traits are implicated in regulatory function of cognition and behavior. Particularly, the absence of behavioral inhibition seems to have a central role, negatively predicting EF. Conversely, IMP positively predicted EF in the model. In fact, scores at the BIS/BAS were differentially indicating poor ability to inhibit response and, at the same time, enhanced reactivity to environmental cues, affecting the performance at the WCST, especially in terms of perseverance in responses.

This model supports findings from neurodevelopmental research (see Romer, 2010). In fact, high reward sensitivity and low behavioral inhibition, considered as major predictors of risk-taking during adolescence (Gullo & Dawe, 2008), revealed valid self-report measures in assessing adolescents’ deficits in regulating responses to conflicting environmental requests and set shifting, as indicated by the WCST performance. Previous neuroimaging studies attributed this pattern to dopaminergic projections from the ventral tegmental area to the nucleus accumbens, determining a lack of IC in response to cues associated with salient stimuli, as in the case of substance use (Gullo & Dawe, 2008).

Yet in our study, the Five-Factor Model of Personality (Aluja et al., 2010) was not significantly related to EF, in line with our theoretical assumptions. Particularly, we found that novelty seeking was not related to cognitive abilities assessed through the WCST performance. In the same vein, previous studies already showed that observed differences in novelty seeking during adolescence were not affecting EF and control over behavior, indicating a temporary rise in the activation of the ventral striatum rather than a structural deficit in frontal control (Romer, 2010).

We consider our results as informative of the validity of self-report measures to examine the relation between IMP and IC traits and neurocognitive function. In fact, in our measurement model, the latent factor IMP explained 41% of the variance of BIS11 and 33% of DII. The latent factor Control explained 65% of the variance of BIS and 63% of BAS.

This study has three main limitations. First, the relations between self-report subscales and latent factors were not assessed, as in the measurement model of SEM we utilized total scores as indicators of latent variables, and accordingly, we were not able to firmly state the contribution of subscales’ scoring to the analyzed dimensions - albeit corresponding to our theoretical concerns and widely described in literature - and to address the associations between such dimensions and single regulatory functions assessed by the WCST performance. Second, self-report measures did not permit to specify *objective* relations and predictions related to the dimensions examined, and results are not exhaustive with certainty. Therefore, we believe future research should be concerned with the study of the fit of self-reports assessments to laboratory and neuroimaging outcomes, examining associations between IMP, IC, and personality with EF. Third, given the cross-sectional design of the study, results are not generalizable, and research employing a panel study with a growth curve model is needed to clarify the predictive power of the model.
